# Continuous venovenous hemodiafiltration with a low citrate dose regional anticoagulation protocol and a phosphate-containing solution: effects on acid–base status and phosphate supplementation needs

**DOI:** 10.1186/1471-2369-14-232

**Published:** 2013-10-25

**Authors:** Santo Morabito, Valentina Pistolesi, Luigi Tritapepe, Elio Vitaliano, Laura Zeppilli, Francesca Polistena, Enrico Fiaccadori, Alessandro Pierucci

**Affiliations:** 1 Department of Nephrology and Urology, Hemodialysis Unit, Umberto I, Policlinico di Roma, “Sapienza” University, Rome, Italy; 2 Department of Anesthesiology and Intensive Care, Cardiac Surgery ICU, Umberto I, Policlinico di Roma, “Sapienza” University, Rome, Italy; 3 Division of Nephrology and Dialysis, Pertini Hospital, Rome, Italy; 4 Department of Clinical and Experimental Medicine, Acute and Chronic Renal Failure Unit, Parma University, Parma, Italy

**Keywords:** AKI, Citrate, CRRT, CVVH, CVVHDF, Hypophosphatemia, Regional citrate anticoagulation

## Abstract

**Background:**

Recent guidelines suggest the adoption of regional citrate anticoagulation (RCA) as first choice CRRT anticoagulation modality in patients without contraindications for citrate. Regardless of the anticoagulation protocol, hypophosphatemia represents a potential drawback of CRRT which could be prevented by the adoption of phosphate-containing CRRT solutions. The aim was to evaluate the effects on acid–base status and phosphate supplementation needs of a new RCA protocol for Continuous Venovenous Hemodiafiltration (CVVHDF) combining the use of citrate with a phosphate-containing CRRT solution.

**Methods:**

To refine our routine RCA-CVVH protocol (12 mmol/l citrate, HCO_3_^-^ 32 mmol/l replacement fluid) (*protocol A*) and to prevent CRRT-related hypophosphatemia, we introduced a new RCA-CVVHDF protocol (*protocol B*) combining an 18 mmol/l citrate solution with a phosphate-containing dialysate/replacement fluid (HCO_3_^-^ 30 mmol/l, Phosphate 1.2). A low citrate dose (2.5–3 mmol/l) and a higher than usual target circuit-Ca^2+^ (≤0.5 mmol/l) have been adopted.

**Results:**

Two historical groups of heart surgery patients (n = 40) underwent RCA-CRRT with *protocol A* (n = 20, 102 circuits, total running time 5283 hours) or *protocol B* (n = 20, 138 circuits, total running time 7308 hours). Despite higher circuit-Ca^2+^ in *protocol B* (0.37 vs 0.42 mmol/l, p < 0.001), circuit life was comparable (51.8 ± 36.5 vs 53 ± 32.6 hours). *Protocol A* required additional bicarbonate supplementation (6 ± 6.4 mmol/h) in 90% of patients while *protocol B* ensured appropriate acid–base balance without additional interventions: pH 7.43 (7.40–7.46), Bicarbonate 25.3 (23.8–26.6) mmol/l, BE 0.9 (-0.8 to +2.4); median (IQR). No episodes of clinically relevant metabolic alkalosis, requiring modifications of RCA-CRRT settings, were observed. Phosphate supplementation was needed in all group A patients (3.4 ± 2.4 g/day) and in only 30% of group B patients (0.5 ± 1.5 g/day). Hypophosphatemia developed in 75% and 30% of group A and group B patients, respectively. Serum phosphate was significantly higher in *protocol B* patients (P < 0.001) and, differently to *protocol A*, appeared to be steadily maintained in near normal range (0.97–1.45 mmol/l, IQR).

**Conclusions:**

The proposed RCA-CVVHDF protocol ensured appropriate acid–base balance without additional interventions, providing prolonged filter life despite adoption of a higher target circuit-Ca^2+^. The introduction of a phosphate-containing solution, in the setting of RCA, significantly reduced CRRT-related phosphate depletion.

## Background

Continuous renal replacement therapy (CRRT) is the most widely adopted technique for the treatment of acute kidney injury (AKI) in the critically ill [1-3] and it is well known that the need for prolonged anticoagulation still represents its main drawback [4-6]. Indeed, although the incidence of bleeding complications in patients undergoing renal replacement therapy (RRT) can be extremely variable among different studies, the risk of major bleeding during standard anticoagulation with heparin should be strongly considered [5,7]. Among different options, regional citrate anticoagulation (RCA) has been increasingly suggested as a safe and efficacious alternative to standard heparin anticoagulation during CRRT [8-19].

Citrate provides anticoagulation in the extracorporeal circuit by chelation of ionized calcium [8], which is required as a key cofactor in several steps of the clotting cascade [20]. A citrate solution is infused before the filter, being the citrate dose titrated to maintain ionized calcium levels in the extracorporeal circuit below 0.3–0.4 mmol/l. Part of the infused citrate is removed by the treatment itself, depending on its operative settings; citrate returning to the patient is rapidly metabolized by the liver and the skeletal muscle in the Krebs’ cycle, with an ensuing bicarbonate production which provides a buffer supply to the patient [8]. On these bases, the citrate metabolic load for the patient is the difference between the delivered dose of citrate and the amount of citrate lost in the effluent [21]. Therefore, different combinations of citrate solutions and replacement fluids for CRRT, as well as the operational parameters setting peculiar of each RRT modality, might be associated with a high variability of buffers supply, thus significantly affecting the acid–base status of the patient [22-24].

Hypophosphatemia is a known issue of CRRT reported in up to 80% of cases when standard CRRT solutions are used [25-30], especially if high dialysis doses are delivered [26,27]. RRT-related phosphate depletion should be avoided in critically ill patients and the adoption of phosphate-containing CRRT solutions could be helpful to reduce the incidence of hypophosphatemia and/or to minimize the need for parenteral phosphorus supplementation [24,25,28,30,31].

In the present study we evaluated the effects on acid–base status and serum phosphate levels of a new RCA protocol for Continuous Venovenous Hemodiafiltration (CVVHDF) using an 18 mmol/l citrate solution in combination with a phosphate-containing solution, acting as dialysate and replacement fluid. The new protocol was introduced with the following targets: a) to refine buffers balance of a previously adopted RCA protocol for Continuous Venovenous Hemofiltration (CVVH), based on a 12 mmol/l citrate solution combined with a conventional replacement fluid; b) to prevent CRRT-related phosphate depletion; c) to maintain a low citrate dose, adopting a higher than usual target circuit ionized calcium.

## Methods

In this observational study, data prospectively collected from May 2010 to December 2012 have been analysed to compare a previously adopted RCA-CVVH protocol with a newly designed RCA-CVVHDF protocol in two historical groups of patients who consecutively underwent CRRT for AKI following major heart surgery at the Cardiac Surgery ICU of Policlinico Umberto I, “Sapienza” University (Rome, Italy). Only patients treated for at least 72 hours have been included in the analysis. The study was in agreement with the Declaration of Helsinki and informed consent was obtained from either the patient or a close relative. Ethics Committee approval was not required for this observational study because the patients, included in a retrospective analysis of prospectively collected data, were not discretionally assigned to different medical interventions. Indeed, in this study we report, in two groups of patients who underwent RCA-CRRT in subsequent historical periods, the effects of the change of our routine RCA protocol after commercial availability of new solutions, registered in our country for specific use in CRRT. At our institution, RCA is the standard anticoagulation method in high bleeding risk heart surgery patients undergoing CRRT and data collection, as well as RCA protocols, are part of our routine medical procedures. Starting from April 2012, according to KDIGO Clinical Practice Guideline for AKI [6] and regardless of the coagulation status, the adoption of RCA was extended to all patients undergoing CRRT without contraindications for citrate.

**Figure 1:**
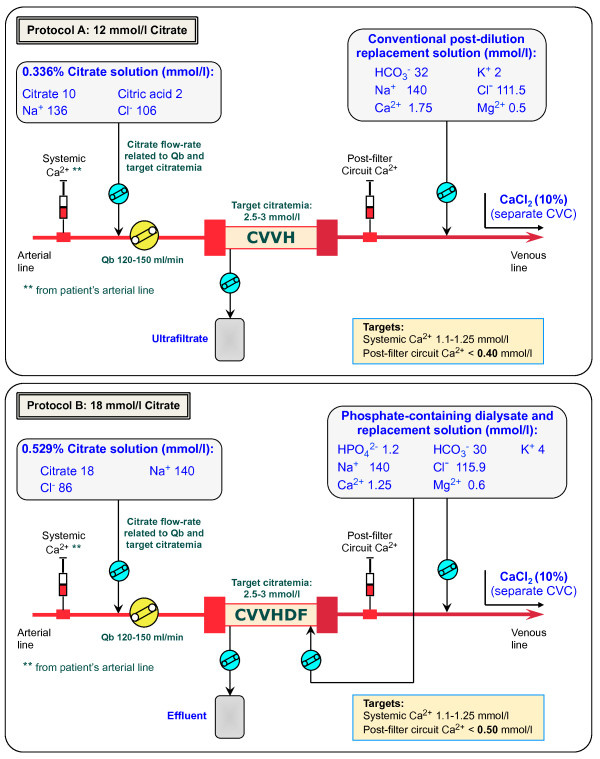
**Pre-post dilution RCA-CVVH and RCA-CVVHDF circuits.** Schematic representation of the RCA extracorporeal circuits reporting the composition of the solutions respectively adopted in *protocol A***(top panel)** and *protocol B***(bottom panel)**.

Until September 2011, RCA was performed in CVVH modality with a 12 mmol/l pre-dilution citrate solution (trisodium citrate 10 mmol/l, citric acid 2, Na^+^ 136, Cl^-^ 106; Prismocitrate 10/2, Gambro, Sondalo, Italy) and a calcium-containing post-dilution replacement fluid with bicarbonate (HCO_3_^-^ 32 mmol/l, Ca^2+^ 1.75, Mg^2+^ 0.5, K^+^ 2, Na^+^ 140, Cl^-^ 111.5; Prismasol 2, Gambro, Sondalo, Italy) (*Protocol A*) (Figure 1) [19]. In case of worsening metabolic acidosis, not related to citrate accumulation and persisting after RCA-CVVH parameters optimization, additional bicarbonate infusion in a separate line was started according to ICU physician’s judgement. In order to optimize buffers balance and to possibly reduce the need for phosphorus supplementation, we implemented a new protocol (*Protocol B*) adopting the following solutions, recently introduced in Europe: 18 mmol/l pre-dilution citrate solution (trisodium citrate 18 mmol/l, Na^+^ 140, Cl^-^ 86; Prismocitrate 18/0, Gambro) combined with a calcium- and phosphate-containing solution, acting as dialysate as well as post-dilution replacement fluid (HPO_4_^2-^ 1.2 mmol/l, HCO_3_^-^ 30, Ca^2+^ 1.25, Mg^2+^ 0.6, K^+^ 4, Na^+^ 140, Cl^-^ 115.9; Phoxilium, Gambro) (Figure 1). The CVVHDF modality has been preferred with the aim to maintain a low filtration fraction. The protocol has been designed through a mathematical model developed to roughly estimate metabolic citrate load, buffers balance (citrate and bicarbonate), effluent calcium loss, as well as the main RCA-CRRT parameters. The model, included in a database software (FileMaker Inc, Santa Clara, CA, USA), and compatible with many portable devices, allowed to easily making calculations at the bedside. Input fields: blood flow rate (ml/min), citrate solution concentration (mmol/l), citrate solution flow rate (l/h), bicarbonate and ionized calcium dialysate and/or replacement solution concentration (mmol/l), dialysate flow rate (l/h), post-dilution flow rate (l/h), patient’s bicarbonate and ionized calcium (mmol/l), patient’s hematocrit (%) and serum protein (g/dl), net ultrafiltration rate (l/h). Calculated output fields (corrected for pre-dilution when appropriate): pre-filter estimated citrate blood concentration (mmol/l) calculated in plasma water *[(citrate solution concentration* × *citrate flow rate)/(citrate flow rate + plasma water flow rate)]*, total effluent rate (l/h), filtration fraction (%), estimated citrate metabolic load (mmol/h) *[(citrate solution concentration* × *citrate flow rate) – (effluent rate* × *estimated citrate blood concentration* × *SC)]*, CRRT buffers and calcium balance (mmol), suggested CaCl_2_ infusion rate (ml/h).

CRRT was performed using the Prismaflex system (Gambro Lundia AB, Lund, Sweden) and PAES hemofilters (HF 1000, 1.15 m^2^, Gambro, Meyzieu, France). Vascular access was obtained by cannulation of the internal jugular or femoral vein with a double lumen polyurethane catheter (⊘ 12 French). In relation to blood flow rate, the citrate solution flow rate was initially set to meet a roughly estimated target circuit citrate concentration of 2.5–3 mmol/l, calculated in plasma water [32,33]. For *protocol A*, citrate flow rate was modified, if needed, to achieve a circuit Ca^2+^ (c-Ca^2+^) ≤0.4 mmol/l. For *protocol B*, taking into account the combination of a more concentrated citrate solution with a 30 mmol/l bicarbonate dialysate/replacement fluid, we accepted a higher than usual c-Ca^2+^ target of ≤0.5 mmol/l with the aim to maintain a low citrate dose and to prevent the occurrence of metabolic alkalosis related to buffer overload. In *protocol B* (CVVHDF), dialysate flow was maintained at the fixed rate of 500 ml/h. Post-dilution flow rate (Prismasol 2 for *protocol A*, Phoxilium for *protocol B*) was adjusted to achieve a prescribed dialysis dose, corrected for pre-dilution *[correction factor = blood flow rate/(blood flow rate + Pre-dilution infusion rate)]*, of at least 25 ml/kg/h. Calcium chloride (10%) was infused in a separate central venous line to maintain a target systemic Ca^2+^ (s-Ca^2+^) of 1.1–1.25 mmol/l, measured by arterial blood gases at least every 4 hours. A total calcium/Ca^2+^ ratio (Calcium Ratio) > 2.5 was considered an indirect sign of citrate accumulation [34]. Serum electrolytes, coagulation parameters and complete blood count were daily assessed.

By convention, hypophosphatemia was defined as follows: mild (<0.81 mmol/l), moderate (<0.61 mmol/l) and severe (<0.32 mmol/l) [30]. Nutritional support was provided mainly via parenteral route associated, if tolerated, with enteral route; energy and protein intake targets were 25 Kcal/Kg/day and 1.5 g/Kg/day with a phosphorus intake of about 20–30 mmol/day during both protocol periods. Potassium, phosphate and magnesium losses with CRRT were replaced, when needed, respectively with potassium chloride, d-fructose-1,6-diphosphate (FDP; Esafosfina® 5 g/50 ml) and magnesium sulphate. In particular, FDP administration was scheduled in case of phosphate levels <0.9 mmol/l. Acid–base parameters, K^+^ and Ca^2+^ were measured by arterial blood gases analyzer (GEM Premiere 4000, Instrumentation Laboratory UK Ltd, Warrington, UK) at least every 4 hours. Clinically relevant metabolic alkalosis was arbitrarily defined as a persistent increase of pH >7.50 and bicarbonate >30 mmol/l.

The causes for CRRT stopping were reported after an accurate evaluation of monitor events and pressure alarms, recorded on the Prismaflex memory card. CRRT interruption due to coagulation was defined as an overt sign of circuit clotting, or as a 100% increase of filter drop pressure (difference between pre-filter and post-filter hydrostatic pressure). CRRT interruption for clinical reasons (i.e., evaluation of renal function recovery, patient mobilization, etc.), unrelated to circuit clotting, was classified and reported as scheduled CRRT stopping.

### Statistical analysis

Data are reported as mean ± standard deviation (SD) or as median and interquartile range (IQR). Statistical analysis for continuous variables was made by one-way ANOVA or Student t-test. Non-parametric analysis was performed, when appropriate, using the Mann–Whitney U test for independent samples. Categorical variables were analysed with chi-square test or Fisher exact test. Circuit lifetime was evaluated with Kaplan-Meier survival analysis and survival curves distribution was compared with the Log Rank (Mantel-Cox) test. All tests were 2-sided (significance level 5%). IBM SPSS statistical software (19.0, SPSS Inc., USA) was used for all analysis.

## Results

Twenty patients underwent RCA-CVVH with *protocol A* while, after introduction of the new protocol, 20 patients were treated with RCA-CVVHDF according to *protocol B*. Clinical characteristics of the patients at the time of starting CRRT have been compared and reported for both groups in Table 1. Among RCA-CRRT initial parameters, reported in Table 2, prescribed dialysis dose, corrected for pre-dilution, as well as citrate dose, were comparable.

**Table 1:** Clinical characteristics of the patients at CRRT start

**Variable**	**Protocol A (n = 20)**	**Protocol B (n = 20)**	**P value**
Female gender	7/20 (35%)	4/20 (20%)	0.480
Age, years	72 (69–77)	70 (59–73)	0.073
Body weight, kg	72 (67–79)	70 (66–80)	0.715
Oliguric AKI ^§^	90%	75%	0.407
Mean arterial pressure, mmHg	70 (70–80)	70 (62–80)	0.399
Use of vasopressors or inotropes	75%	85%	0.695
Mechanical ventilation	85%	90%	0.633
Artificial nutrition	95%	100%	0.311
APACHE II score	32 (29–35)	32 (27–35)	0.307
SOFA score	15 (12–16)	13 (9–14)	0.056
SOFA cardio-vascular score	3 (2–3)	3 (2–3)	0.130
Serum creatinine, mg/dl	2.10 (1.75–3.00)	2.25 (1.75–2.85)	0.469
Blood urea nitrogen, mg/dl	40.5 (26.9–62.0)	40.5 (29.0–55.2)	0.978
Hemoglobin, g/dl	10.9 (9.9–11.2)	10.0 (8.8–10.9)	0.084
Hematocrit,%	32.1 (31.2–33.4)	31.6 (27.5–34.5)	0.419
White blood cells, ×10^3^/μl	11.0 (8.2–15.9)	12.4 (11.0–16.5)	0.664
Platelet count, ×10^3^/μl	93 (76–177)	161 (98–254)	0.043
Antithrombin III activity,%	70 (63–78)	61 (49–78)	0.250
APTT Ratio	1.4 (1.3–1.9)	1.6 (1.4–2)	0.358
Sodium, mmol/l	139 (134–140)	139 (137–143)	0.110
Potassium, mmol/l	4.3 (4.0–4.8)	4.2 (4.1–4.8)	0.762
Total Calcium, mmol/l	2.16 (1.95–2.30)	2.03 (1.93–2.19)	0.166
Phosphorus, mmol/l	1.40 (1.07–1.58)	1.38 (1.16–1.70)	0.897
Magnesium, mmol/l	0.85 (0.75–0.93)	0.78 (0.74–0.97)	0.572
pH, units	7.38 (7.35–7.41)	7.40 (7.38–7.43)	0.324
Bicarbonate, mmol/l	21.8 (21.0–22.6)	22.2 (21.0–24.2)	0.535
Base Excess	-3.5 (-4.0 to -2.0)	-1.0 (-4.0 to 1.0)	0.316
pCO_2_, mmHg	38 (36–42)	37 (33–40)	0.992
Lactate, mmol/l	1.4 (1.0–3.0)	1.4 (1.2–2.4)	0.879
Bilirubin, mg/dl	0.89 (0.62–1.45)	0.73 (0.46–1.37)	0.376
Aspartate aminotransferase, IU/l	89 (29–608)	97 (49–376)	0.800
Alanine aminotransferase, IU/l	37 (12–325)	26 (18–75)	0.970
Albumin, g/dl	2.6 (2.4–2.8)	2.45 (2.2–2.9)	0.820
**Heart surgery:**			**Overall**
Coronary artery bypass grafting	25%	35%	30%
Coronary artery bypass grafting + valvular surgery	40%	15%	27.5%
Valvular surgery	25%	20%	22.5%
Ascending aorta replacement	10%	30%	20%

Data are expressed as median (IQR) or percentage. ^§^ According to AKIN criteria (Crit Care 2007; 11:R31).

**Table 2:** Initial RCA-CRRT settings

**RCA-CRRT settings**	**Protocol A**	**Protocol B**	**P-value**
Prescribed dialysis dose^§^, ml/kg/h	28.25 (26.69–29.66)	26.67 (25.69–28.88)	0.333
Blood flow rate, ml/min	130 (130–140)	140 (140–140)	0.083
Pre-dilution citrate solution flow rate, l/h	1.56 (1.50–1.68)	1.00 (1.00–1.00)	<0.001
Pre-dilution,%	16.67 (16.58–16.67)	10.64 (10.64–10.64)	<0.001
Post-dilution replacement fluid flow rate, l/h	0.80 (0.80–0.95)	0.60 (0.55–0.80)	<0.001
Dialysate flow rate, l/h	N/A	0.50 (0.50–0.50)	-
Filtration Fraction,%	38.4 (34.5–40.3)	27.0 (25.7–28.8)	<0.001
10% Calcium chloride infusion rate, mmol/h	2.00 (1.60–2.10)	1.90 (1.36–2.28)	0.344
Citrate infusion rate, mmol/h	18.70 (18.00–20.15)	18.00 (18.00–18.00)	0.051
Estimated citrate load, mmol/h	12.34 (11.74–13.92)	12.35 (12.05–12.65)	0.471
Estimated citrate dose, mmol/l	2.84 (2.74–2.91)	2.83 (2.76–2.95)	0.618

Data are expressed as median (IQR). ^§^ Corrected for pre-dilution.

One hundred and two circuits (total running time 5283 hours) were used during *protocol A* period while 138 circuits (total running time 7308 hours) were used after adoption of *protocol B*. Filter life was 51.8 ± 36.5 and 53 ± 32.6 hours, respectively (P = 0.796) (Table 3).

**Table 3:** Circuit lifetime and CRRT interruption causes

	**Protocol A (n = 102)**	**Protocol B (n = 138)**	**Overall (n = 240)**
**CIRCUIT LIFETIME**			
Mean ± SD, h	51.8 ± 36.5	53 ± 32.6	**52.5 ± 34.2**
Median (IQR), h	44.5 (24.0–72.0)	47.5 (24.0–78.5)	**47.5 (24.0–75.0)**
> 24 h	77%	76%	**76.7%**
> 48 h	50%	50%	**50%**
> 72 h	28%	33%	**31.2%**
**CRRT STOPPING CAUSES**			
CVC malfunction, n (%)	40 (39.2%)	49 (35.5%)	**89 (37.1%)**
Alarm handling/ technical issues, n (%)	26 (25.4%)	31 (22.5%)	**57 (23.8%)**
Scheduled, n (%)	24 (23.6%)	36 (26.1%)	**60 (25%)**
Medical procedures, n (%)	12 (11.8%)	13 (9.4%)	**25 (10.4%)**
Clotting, n (%)	0 (0%)	9 (6.5%)	**9 (3.7%)**

Data are expressed as mean ± SD or median (IQR) or percentage.

**Figure 2:**
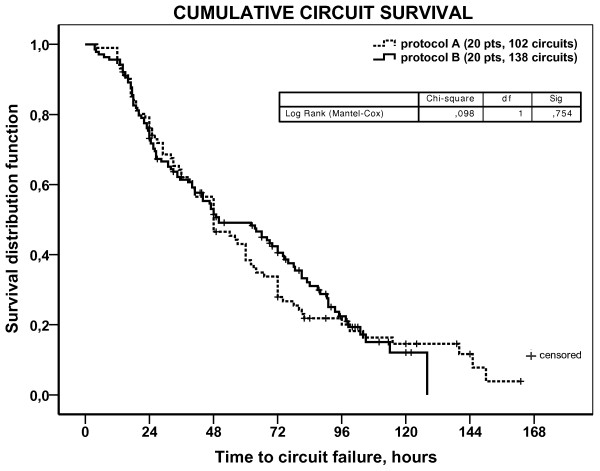
**Kaplan-Meier curves of circuit lifetime probability according to the two RCA protocols.** Survival curves**,** derived from the analysis of scheduled and unscheduled CRRT stopping for any cause, have been compared with the Log Rank (Mantel-Cox) test (p = 0.754).

RCA-CRRT stopping causes and circuits running at 24, 48, 72 hours are reported in Table 3. Considering all circuits (n = 240), filter clotting was the less frequent cause for RCA-CRRT interruption (3.7%). In particular, during *protocol A*, RCA-CVVH didn’t stop in any case for filter clotting while 6.5% of *protocol B* CRRT sessions were stopped for significant increments of filter drop pressure (>100%) (P = ns). Overall, pressure alarms handling, related to CVC malfunction, was the most frequent cause of CRRT stopping (37.1%). Kaplan-Meier curves of circuit lifetime probability, derived from analysis of scheduled and unscheduled CRRT stoppings for any cause, showed no difference between the two protocols (Figure 2).

**Figure 3:**
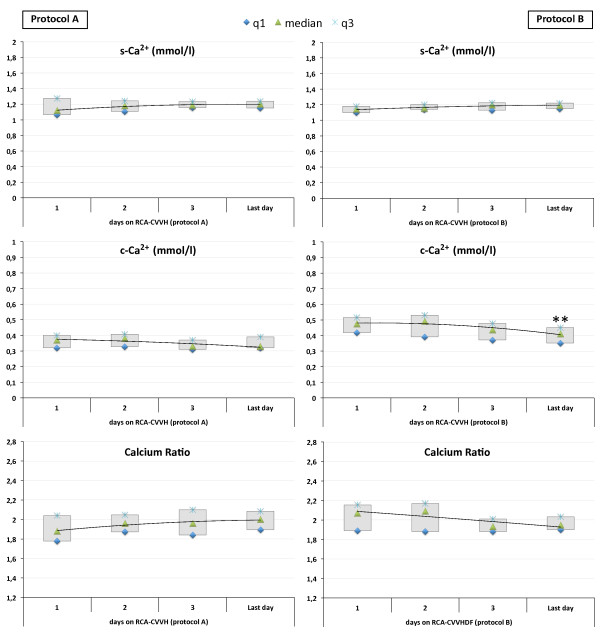
**Systemic ionized calcium (s-Ca**^**2+**^**), circuit ionized calcium (c-Ca**^**2+**^**) and Calcium Ratio throughout RCA-CRRT days with the two different protocols.** Data for *protocol A***(left panels)** and *protocol B***(right panels)** are displayed as median and interquartile range (q1 to q3). ** p < 0.02.

Calcium monitoring parameters, including c-Ca^2+^, s-Ca^2+^ and Calcium Ratio for each patient at different treatment days, are displayed in Figure 3. Circuit Ca^2+^ was maintained in the target range adopted for *protocol A* and *protocol B* and was significantly higher during RCA-CVVHDF with *protocol B* (median 0.37 vs 0.42 mmol/l, P < 0.001) (Table 4). With both protocols, s-Ca^2+^ was steadily maintained in normal range with few modifications of CaCl_2_ flow rate (1–2 within 24 hours), without episodes of hypocalcemia or hypercalcemia (Figure 3). The amount of CaCl_2_ required to maintain s-Ca^2+^ in the target range was comparable (P = 0.800) (Table 4).

**Table 4:** Laboratory variables and supplementation needs during RCA-CRRT

**Variable**	**Protocol A (n = 20)**	**Protocol B (n = 20)**	**P value**
Systemic Ca^2+^, mmol/l	1.16 (1.10–1.23)	1.16 (1.11–1.21)	0.995
Total Calcium, mmol/l	2.33 (2.20–2.50)	2.33 (2.21–2.45)	0.474
Calcium Ratio	1.95 (1.84–2.09)	1.98 (1.89–2.09)	0.296
Circuit Ca^2+^, mmol/l	0.37 (0.32–0.40)	0.42 (0.36–0.47)	<0.001
Sodium, mmol/l	134 (133–136)	134 (132–135)	0.213
Potassium, mmol/l	4.2 (4.0–4.3)	4.2 (4.0–4.4)	0.159
Phosphate, mmol/l	0.70 (0.50–1.00)	1.20 (0.97–1.45)	<0.001
Magnesium, mmol/l	0.78 (0.58–0.95)	0.79 (0.73–0.84)	<0.001
pH, units	7.40 (7.36–7.44)	7.43 (7.40–7.46)	<0.001
Bicarbonate, mmol/l	22.1 (20.9–23.5)	25.3 (23.8–26.6)	<0.001
Base Excess	-3.1 (-4.6 to -1.1)	0.9 (-0.8 to 2.4)	<0.001
pCO_2_, mmHg	35 (32–41)	38 (35–40)	0.527
Apparent strong ion difference (AppSID), mEq/l	37.6 (36.1–39.4)	39.1 (37.9–40.2)	<0.001
Effective strong ion difference (EffSID), mEq/l	32.0 (30.6–33.1)	34.3 (32.8–35.9)	<0.001
Strong ion gap (SIG), mEq/l	5.8 (3.5–7.7)	4.5 (3.1–6.9)	0.585
Lactate, mmol/l	1.2 (0.9–1.6)	1.1 (0.8–1.4)	<0.001
Platelet count, ×10^3^/μl	90 (53–156)	187 (121–261)	<0.001
Antithrombin III activity,%	72 (62–81)	75 (63–89)	0.078
aPTT Ratio	1.4 (1.3–1.6)	1.4 (1.2–1.7)	0.575
**Supplementation needs**			
CaCl_2_ infusion, mmol/h	2.18 (1.90–2.45)	2.24 (1.97–2.58)	0.800
KCl infusion, mmol/h	5 (3–7)	2 (0–4)	<0.001
Magnesium Sulphate, g/day	3 (2–3)	3 (3–3)	<0.001
Need for bicarbonate infusion, n (%)	18/20 (90%)	0/20 (0%)	<0.001
Infusion rate, mmol/h	6 ± 6.4	No supplementation	
Need for phosphate supplementation, n (%)	20/20 (100%)	6/20 (30%)	<0.001
g of phosphorus/day	3.39 ± 2.45	0.52 ± 1.53	

Data are expressed as median (IQR) or mean ± SD or percentage.

**Figure 4:**
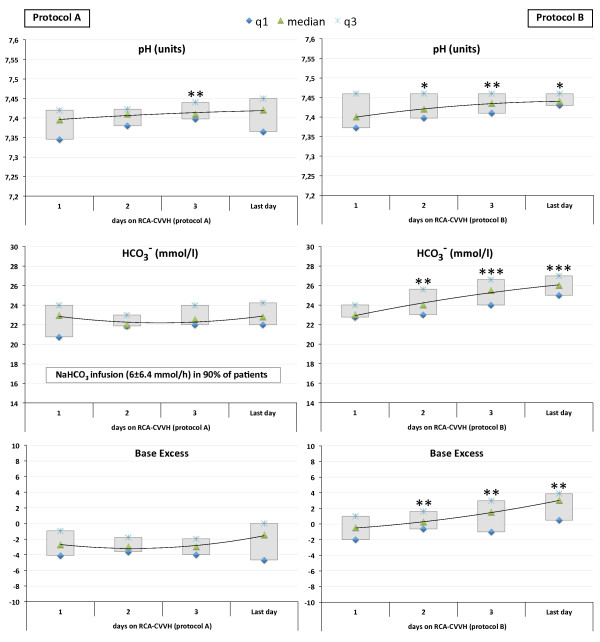
**Main acid–base parameters throughout RCA-CRRT days with the two different protocols.** Data for *protocol A***(left panels)** and *protocol B***(right panels)** are displayed as median and interquartile range (q1 to q3). * p < 0.05, ** p < 0.02, *** p < 0.001.

Acid–base parameters and the main serum electrolytes for both groups of patients are reported in Table 4. Serum bicarbonate levels and pH values were significantly higher in *protocol B* patients (P < 0.001), without the need for additional bicarbonate infusion, which was otherwise required in 18 out of 20 *protocol A* patients (NaHCO_3_ infusion rate 6 ± 6.4 mmol/h). No episodes of clinically relevant metabolic alkalosis, requiring additional intervention on RCA-CRRT settings, were observed with both protocols. In particular, during *protocol B*, pH values resulted ≥7.50 in 3.8% of determinations (63 out of 1664) with a Base Excess ≥5 in 3.2% of determinations (54 out of 1664). Acid–base parameters throughout RCA-CRRT days, including values of pH, bicarbonate and Base Excess for each patient at different treatment days, are displayed in Figure 4. Regardless of the RCA-CRRT protocol, no episodes of metabolic acidosis, possibly related to inadequate citrate metabolism, were observed and Calcium Ratio resulted constantly below the accepted threshold value of 2.5.

**Figure 5:**
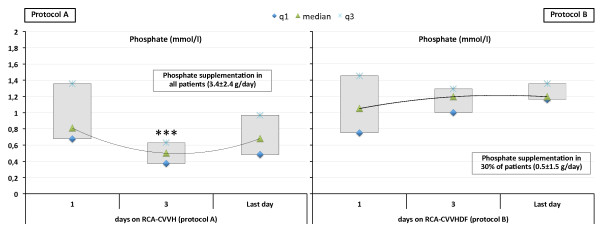
**Serum phosphate and need for phosphorus supplementation throughout RCA-CRRT days with the two different protocols.** Data for *protocol A***(left panel)** and *protocol B***(right panel)** are displayed as median and interquartile range (q1 to q3). *** p < 0.001.

At some times during RCA-CRRT, 75% of *protocol A* patients developed hypophosphatemia (6 mild, 9 moderate), otherwise observed in only 30% of *protocol B* patients (4 mild, 2 moderate) (P < 0.001). In particular, 89 out of 206 phosphorus level determinations met the definition criteria for mild to moderate hypophosphatemia during *protocol A* (42 mild, 47 moderate) while a significantly lower number of determinations (26 out of 334; P < 0.001) revealed episodes of hypophosphatemia during *protocol B* (19 mild, 7 moderate). *Protocol A* required phosphorus supplementation (FDP 3.39 ± 2.45 g/day) in all patients. A lower amount of phosphorus supplementation (FDP 0.52 ± 1.53 g/day) was needed in 6 out of 20 patients (30%) undergoing *protocol B*. Serum phosphate was significantly higher in *protocol B* patients (P < 0.001) and, differently to *protocol A*, it appeared to be steadily maintained in near normal range (IQR 0.97–1.45 mmol/l) without episodes of hyperphosphatemia requiring modifications of CRRT settings (Table 4). Serum phosphate levels throughout RCA-CRRT days are displayed in Figure 5 for both protocols.

Clinically relevant hypomagnesemia has been prevented in all patients by magnesium sulphate supplementation (2 to 3 g/day) (Table 4). Serum potassium was steadily maintained in normal range with both protocols. As expected, the need for potassium chloride supplementation was significantly lower with *protocol B* (P < 0.001) (Table 4).

During RCA-CRRT no patients had bleeding complications and overall transfusion rate was 0.33 ± 0.23 blood units/day (median 0.24, IQR 0.18–0.43), without differences between the two groups (0.32 ± 0.2 versus 0.33 ± 0.26, P = 0.704). Overall, thirty-day survival was 67.5% while survival at discharge from the hospital was 57.5%. At the time of discharge, renal function recovery, allowing to stop RRT, was observed in 9 out of 11 survivors of protocol A patients (81.8%) and in 10 out of 12 survivors of protocol B patients (83.3%).

## Discussion

The need for continuous anticoagulation represents a potential drawback of CRRT modalities [4-6]. Recently published guidelines suggest the adoption of RCA as first choice CRRT anticoagulation modality in patients without contraindications for citrate, especially in the presence of increased bleeding risk [6].

At our institution, RCA is now routinely adopted in heart surgery patients undergoing CRRT with the aim to minimize bleeding complications. In the present study, both RCA protocols allowed to maintain low transfusion rates in a small cohort of selected high bleeding risk patients, ensuring an adequate filter life with a very low incidence of clotting as cause of CRRT stopping (9 out of 240 CRRT sessions). In particular, the use of a low flow rate calcium-containing dialysate, which characterizes protocol B, didn’t appear to adversely affect mean filter life. Regarding the clotting events observed in a small proportion of protocol B circuits, it is tempting to speculate that this finding could be explained by the strategy to allow higher levels of Ca^2+^ inside the circuit, although the possible role of the significantly higher platelet count should be considered. On the other side, this potential drawback of protocol B could be counterbalanced by the advantage of a more easy maintenance of a low citrate dose (2.5–3 mmol/l in plasma water). In this regard, taking into consideration that any strategy aimed to prevent citrate accumulation should be targeted to decrease citrate infusion rate [35], both protocols have been designed with the aim to minimize citrate load, with a resulting citrate dose among the lowest until now reported. Regardless of the RCA protocol adopted, no episodes of high anion gap metabolic acidosis, possibly related to inadequate citrate metabolism, were observed. Indeed, the Calcium Ratio, commonly accepted as a useful index of citrate accumulation [34,36,37] and recently reported as an independent predictor of clinical outcome [38], resulted steadily below the conventional threshold value of 2.5 in all patients. On the other hand, the buffers mass balance obtained with *protocol A*, based on a 12 mmol/l citrate solution, was frequently associated with a suboptimal buffers supply despite optimization of RCA-CVVH operative parameters. For this reason, additional bicarbonate infusion was required in most of the patients (90%). Thus, in our experience, the combination of a very low citrate concentration solution (10 mmol/l trisodium citrate, 2 mmol/l citric acid) with a conventional bicarbonate replacement fluid (32 mmol/l) does not allow to tailor buffer delivery according to patient’ needs. This problem cannot be easily overcome by an increase of pre-dilution citrate flow rate, which invariably results in a significant increase of effluent rate with the consequent loss of more citrate and bicarbonate in the ultrafiltrate. Thus, any increment in citrate dose does not result in a clinically significant increment of buffers delivery to the patient. Comparable findings, regarding the persistence of mild metabolic acidosis and the need for additional bicarbonate, have been reported by Hetzel et al., performing CVVH with a 13 mmol/l citrate solution [18], and by Shum et al., adopting CVVH with a 12 mmol/l citrate solution combined with pre-filter infusion of a highly concentrated bicarbonate solution (8.4%) to obtain a more positive buffers balance [39]. However, other authors obtained an appropriate acid–base balance with the use of a slightly higher concentration citrate solution (13.3 mmol/l), without the need of additional bicarbonate infusion [40,41].

In the present study, the adoption of an 18 mmol/l citrate solution (*protocol B*) allowed to more efficiently meet patient’ buffer requirements maintaining, at the same time, a citrate load comparable to *protocol A*. Indeed, in the *protocol B* patients, acid–base status was adequately maintained without additional interventions and both serum bicarbonate levels and pH values were significantly higher to that achieved, despite bicarbonate infusion, in patients undergoing RCA-CVVH with *protocol A*.

The use of an 18 mmol/l citrate solution is not new and has been previously introduced in CVVHDF by Tolwani et al. [22]. Comparing two different citrate solutions during RCA, with the aim to optimize buffers mass balance, the Authors found that the adoption of a citrate concentration of 23 mmol/l was associated with a high incidence of metabolic alkalosis (18 out of 24 patients) while the use of an 18 mmol/l solution was able to provide an appropriate acid–base balance in most of the patients, although at some point during CRRT alkalosis still occurred in 9 out of 32 patients. However, both protocols required a custom-made dialysate with a lower than usual bicarbonate concentration (25 mmol/l) [22]. These findings raised our attention about the risk of alkalosis, possibly enhanced by the standard bicarbonate CRRT solution (30 mmol/l) adopted in *protocol B* as dialysate and post-dilution replacement fluid. Therefore, in order to prevent buffers overload related to the combination of the solutions adopted, we accepted a higher than usual c-Ca^2+^ target (≤0.5 mmol/l) with the aim to minimize the need for any increment of citrate dose throughout RCA-CRRT days. Thus, despite initial concerns, *protocol B* afforded an appropriate acid–base balance without occurrence of clinically significant metabolic alkalosis. In summary, although these findings need to be confirmed in a more consistent number of patients and in a wider range of clinical situations, the solutions combination adopted in *protocol B* appears to be at low risk of acid–base derangements and represents, in our opinion, a step forward if compared to *protocol A*.

By shifting to *protocol B*, our purpose was also to simplify RCA-CRRT handling and to reduce the need for additional infusions. In particular, we aimed at minimizing CRRT-induced phosphate depletion [42] through the combination of the citrate solution with a recently introduced, commercially available, phosphate-containing CRRT solution, acting as dialysate and post-dilution replacement fluid. In this regard, although the prognostic significance of mild to moderate serum phosphate disturbances in critically ill patients is still a matter of discussion [43], it is well known that severe hypophosphatemia can cause generalized muscle weakness and even paralysis of the respiratory muscles, myocardial dysfunction, reduced peripheral vascular resistance and encephalopathy [44]. Therefore, in patients undergoing CRRT, it could be appropriate to prevent hypophosphatemia by addition of phosphate to the replacement and/or dialysate solutions. To this purpose, the feasibility and safety of phosphate addition to conventional dialysate and replacement fluids have been successfully tested in adult and pediatric patients undergoing CRRT [25,28]. More recently, the efficacy of a commercially available phosphate-containing solution in preventing hypophosphatemia has been reported in patients undergoing CVVHDF [30] or CVVH [31]. However, Chua et al. underlined that the switch to a phosphate-containing solution as the sole replacement fluid for CVVH could contribute to mild hyperphosphatemia and could be associated with metabolic acidosis, possibly related to fluid composition [32]. In our RCA-CVVHDF protocol, we adopted the same phosphate-containing solution but it accounted for about 50% of total CRRT dose and, as discussed above, its adoption in combination with an 18 mmol/l citrate solution was not associated with acid–base derangements. Furthermore, as intended, the introduction of the phosphate-containing solution appeared effective to prevent hypophosphatemia in 70% of *protocol B* patients; in the remaining cases, the occurrence of a mild to moderate hypophosphatemia was easily overwhelmed by an amount of phosphorus supplementation much lower than that constantly required in all patients undergoing RCA-CVVH with *protocol A*. In addition, serum phosphate was significantly higher in *protocol B* patients and appeared to be steadily maintained in a narrower range throughout the entire RCA-CVVHDF treatment period without occurrence of clinically relevant hyperphosphatemia. On the contrary, as documented by the presence of hypophosphatemia in more than 40% of determinations, the strategy of parenteral phosphorus supplementation adopted for *protocol A* was associated with wide-ranging variations of phosphatemia during RCA-CVVH. These findings confirm the efficacy of phosphate-containing solutions in reducing the incidence of CRRT-induced hypophosphatemia, already reported elsewhere during conventional CRRT [25,28,30,31], adding useful information about its use in the context of RCA and extending the results of a single-case preliminary experience recently reported by our own group [24]. Lastly, in comparison to most of the protocols reported elsewhere, the adoption of a calcium-containing CRRT solution, which characterizes both protocols, allowed us to reduce CaCl_2_ infusion requirement and to minimize the risk of errors in bags handling related to the use of “zero” calcium solutions.

## Conclusions

In conclusion, the proposed RCA-CVVHDF protocol, using an 18 mmol/l citrate solution, provided a more adequate control of acid–base status if compared to the previously adopted 12 mmol/l RCA-CVVH protocol. The adoption of a low citrate dose, particularly useful in patients with high severity scores, and the maintenance of a higher than usual target circuit Ca^2+^ were still associated with an adequate circuit lifetime and with a very low incidence of clotting as cause of CRRT stopping. Finally, the novel adoption of a phosphate-containing solution, in the setting of RCA, allowed to prevent CRRT-induced hypophosphatemia in most of the patients, minimizing the need for phosphorus supplementation.

## Competing interests

The authors declare that they have no competing interests.

## Pre-publication history

The pre-publication history for this paper can be accessed here:

http://www.biomedcentral.com/1471-2369/14/232/prepub
